# LIFETIME ABUSE VICTIMIZATION AND PROSPECTIVE HEALTH OUTCOMES IN OLDER ADULTS

**DOI:** 10.1093/geroni/igae098.0575

**Published:** 2024-12-31

**Authors:** Jooyoung Kong, Sara Moorman, Yue Qin

**Affiliations:** University of Wisconsin Madison, Madison, Wisconsin, United States; Boston College, Chestnut Hill, Massachusetts, United States; University of Wisconsin Madison, Madison, Wisconsin, United States

## Abstract

Lifetime abuse victimization indicates cumulative violence exposures or repeated victimizations within and/or across different life stages. The current study aims to examine the patterns of lifetime abuse victimization that span childhood to older adulthood and evaluate the associations between lifetime abuse victimization and prospective health outcomes in late adulthood. Data were drawn from 4907 older adults (mean age = 80) from the Wisconsin Longitudinal Study. We assessed the exposure to interpersonal violence including childhood abuse and neglect, intimate partner violence, and elder abuse until the respondents’ age of early 70s. A series of multivariate analyses were conducted to examine the associations of a cumulative score of lifetime abuse victimization with depression, physical health status, and memory in respondents’ age of early 80s. On average, respondents experienced 1.1 exposure to lifetime abuse victimization (SD = 1.53). Greater exposure to lifetime abuse was associated with a significantly higher risk of depression and a greater number of limitations in physical functioning, but not with memory performance. When examined separately by abuse type, childhood abuse was associated with more health limitations and elder abuse was associated with a higher risk of depression and more health limitations. Our results support the interrelations of interpersonal violence across the life course and the lasting health effects of exposure to lifetime abuse, which can lead researchers and practitioners to adopt a more comprehensive conceptualization of interpersonal violence victimization. Findings also highlight the need for a life course-based, trauma-informed approach in prevention and intervention programs for older adults.

